# Arginine Metabolism in Myeloid Cells Shapes Innate and Adaptive Immunity

**DOI:** 10.3389/fimmu.2017.00093

**Published:** 2017-02-07

**Authors:** Paulo C. Rodriguez, Augusto C. Ochoa, Amir A. Al-Khami

**Affiliations:** ^1^Augusta University, Georgia Cancer Center, Augusta, GA, USA; ^2^Stanley S. Scott Cancer Center, Louisiana State University Health Sciences Center, New Orleans, LA, USA; ^3^Department of Pediatrics, Louisiana State University Health Sciences Center, New Orleans, LA, USA; ^4^Department of Genetics, Louisiana State University Health Sciences Center, New Orleans, LA, USA

**Keywords:** arginine, nitric oxide synthase, arginase, immune response, M1 and M2, macrophage, dendritic cell, MDSC

## Abstract

Arginine metabolism has been a key catabolic and anabolic process throughout the evolution of the immune response. Accruing evidence indicates that arginine-catabolizing enzymes, mainly nitric oxide synthases and arginases, are closely integrated with the control of immune response under physiological and pathological conditions. Myeloid cells are major players that exploit the regulators of arginine metabolism to mediate diverse, although often opposing, immunological and functional consequences. In this article, we focus on the importance of arginine catabolism by myeloid cells in regulating innate and adaptive immunity. Revisiting this matter could result in novel therapeutic approaches by which the immunoregulatory nodes instructed by arginine metabolism can be targeted.

## Introduction

Arginine (R) is considered a non-essential amino acid for healthy adult humans since it is endogenously synthesized from the amino acid citrulline as an immediate precursor in virtually all cell types. The small intestine is the major source of citrulline for arginine synthesis by the proximal tubules of the kidneys, known as the intestinal–renal axis for arginine synthesis (1, 2). The normal range of arginine in serum fluctuates between 50 and 150 µM (3, 4). However, arginine is generally classified as a semi or conditionally essential amino acid owing to the fact that arginine must be supplied in the diet in some pathological conditions, including sepsis, trauma, and cancer (5, 6). Arginine metabolism is regulated both through the expression of the y^+^ system of cationic amino acid transporters (7) and through the enzymes responsible for its catabolism. Arginine is metabolized intracellularly by nitric oxide synthase (NOS), arginase, arginine:glycine amidinotransferase (AGAT), and arginine decarboxylase (ADC). These enzymes are expressed in a tissue-specific manner, and some of them are induced under particular inflammatory settings.

Arginine metabolism has emerged as a critical regulator of innate and adaptive immune responses. The major arginine-catabolizing enzymes involved in inflammatory immune responses are the isoforms of NOS (NOS1–3) and arginase (arginase 1 and 2). It is becoming increasingly clear that cells of the myeloid lineage can augment or diminish the immune response *via* the differential regulation of these enzymes. These processes are fundamentally driven by a multitude of inflammatory cues within tissue microenvironments. Importantly, targeting arginine metabolism can modulate key aspects of these critical cells, resulting in a better disease control. As such, this review will discuss how arginine catabolic pathways can lead to heterogeneous, but often opposing, functional consequences and how these mechanisms can be harnessed for the treatment of multiple pathological conditions.

## Arginine Metabolism: NOS

**Figure 1:**
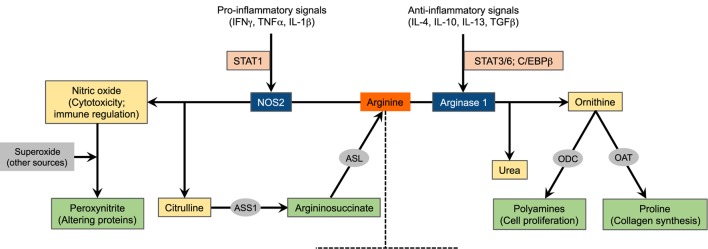
**Schematic of arginine metabolism**. For the sake of simplicity, the major arginine-catabolizing enzymes involved in inflammatory immune responses, NOS2 and arginase 1, are depicted. The expression of these enzymes is tightly regulated by microenvironmental inflammatory signals. This diagram, however, does not suggest that these enzymes are concurrently induced in a given cell type. NOS2, nitric oxide synthase 2; ASS1, argininosuccinate synthase 1; ASL, argininosuccinate lyase; ODC, ornithine decarboxylase; OAT, ornithine aminotransferase.

Three NOS isozymes, encoded by distinct genes, have been identified: NOS1 (known as neuronal NOS, nNOS), NOS2 (known as inducible NOS, iNOS, found in several myeloid cell populations and some T cell subsets), and NOS3 (known as endothelial NOS, eNOS) (2, 8, 9). All NOS enzymes metabolize arginine to produce nitric oxide, which crucially participates in processes associated with vasodilatation and cytotoxic mechanisms (9–11), in addition to citrulline generated as a byproduct. Both NOS1 and NOS3 are constitutively expressed in various types of cells, with their activities being dependent on calcium-calmodulin. On the contrary, NOS2 is controlled through inducible transcription in response to pro-inflammatory cytokines such as interferon γ (IFNγ), tumor necrosis factor α (TNFα), and IL-1β and bacterial lipopolysaccharide (Figure 1). Once stimulated, NOS2 is constantly activated and not controlled by calcium levels (8, 12, 13). Induction of NOS2 has been described primarily in macrophages (14) but also in other cells, including colon (15) and lung (16) epithelial cells and CD4^+^ T cells (17). NOS-derived nitric oxide can stimulate multiple enzymes and proteins inside the target cell. Among these pathways, activation of soluble guanylyl cyclase by nitric oxide to generate cyclic guanosine monophosphate is thought to be the most important (12). NOS is inhibited endogenously by asymmetric dimethylarginine (aDMA), an arginine analog and naturally occurring product of metabolism, or pharmacologically by arginine analogs such as l-NG-monomethylarginine (l-NMMA) among several others (18).

## Arginine Metabolism: Arginase

Arginine is alternatively metabolized by arginases to produce ornithine and urea. Ornithine is the precursor for the production of polyamines *via* the ornithine decarboxylase (ODC) pathway as well as for the production of proline *via* the enzyme ornithine aminotransferase (OAT). While polyamines essentially regulate cell proliferation and differentiation, proline is critical for the synthesis of collagen, a primary protein in wound healing (2, 19, 20). Additionally, urea represents an important mechanism for detoxification of protein degradation. Arginase exists in two isoforms, arginase 1 and arginase 2, that induce the same reaction but are encoded by separate genes and differ in tissue distribution and intracellular localization. Arginase 1 is found in the cytosol of hepatocytes, macrophages, and other myeloid cells and in the granular compartment of human granulocytes. Recently, arginase 1 has been shown to be expressed by mouse and human innate lymphoid cells group 2 (21). Arginase 2, on the other hand, is a mitochondrial enzyme that is expressed in tissues such as kidneys, small intestine, and brain, in addition to most cells in the body (22, 23). The expression of arginase 1 is induced in myeloid cells by the T helper 2 (Th2) cytokines IL-4 and IL-13 (Figure 1). These cytokines activate the signal transducer and activator of transcription 6 (STAT6) that, with other transcription factors such as STAT3 and CCAAT/enhancer binding protein β (C/EBPβ), binds to an enhancer in the arginase 1 locus (24–26). Multiple other factors also induce the expression of arginase 1, including IL-10 (27), granulocyte-macrophage colony-stimulating factor (GM-CSF) (28), transforming growth factor β (TGFβ) (29), prostaglandin E2 (PGE2) (30), cyclic adenosine monophosphate (cAMP) (31), and toll-like receptor (TLR) agonists (32). Arginase 1 expression is also controlled by peroxisome proliferator-activated receptor transcription factors (33, 34). Conversely, arginase 2 is constitutively expressed. Given that the role of myeloid cell arginase 2 in shaping immune responses is not as clearly defined as arginase 1, we will only discuss the latter herein. However, recent evidence indicates that arginase 2 induced in other cell types like asthmatic airway epithelium (35) and activated T cells (36) regulates arginine flux, thereby redirecting the immune response and disease manifestation. This suggests that further investigation of arginase 2 in myeloid cells is warranted.

## NOS and Arginase: Competitors for Arginine

**Figure 2:**
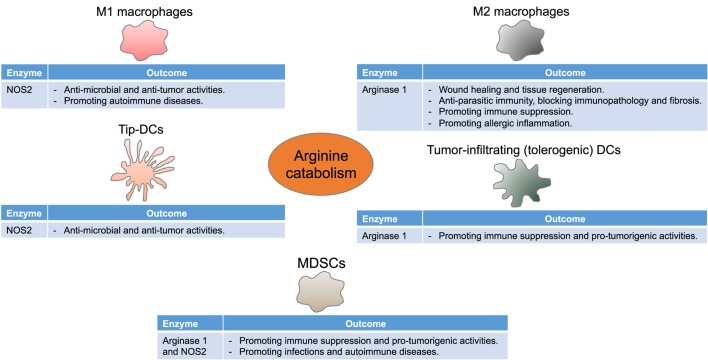
**Arginine metabolism instructs myeloid cells to control immune responses**. Myeloid cells differentially express NOS2 and arginase 1, thereby driving diverse, although seeming contradictory, immune and functional consequences in multiple disease settings. Tip-DCs, tumor necrosis factor α and inducible NOS-producing dendritic cells; MDSCs, myeloid-derived suppressor cells.

Before we discuss the functional consequences of the regulated arginine metabolism in myeloid cells (Figure 2), it is important to emphasize the competition between NOS and arginase for the available intracellular arginine as a major mechanism that dictates the ultimate immune response outcome, as detailed below. The intracellular levels of arginine are in the range of 100–800 µM. The arginine *Km* of NOS is 3 µM, whereas that of arginase is close to 2 mM (2). Therefore, under physiological conditions, NOS should have a higher access to arginine than arginase. However, the *V*_max_ of NOS is almost 1,000 times less than that of arginase, which equilibrates their capabilities to metabolize arginine (2, 37). As a proof of the balanced access of NOS and arginase to arginine is the fact that, despite the higher affinity of NOS to arginine, the production of nitric oxide depends of the extracellular levels of arginine (termed as arginine paradox) (38, 39). A potential explanation for this effect is the subcellular compartmentalization of arginine. However, studies that have regulated the localization of NOS and arginase have failed to confirm this concept (40). An additional level of complexity in the interaction between NOS and arginase is the uncoupling of NOS by arginase (41, 42). The uncoupled NOS produces less nitric oxide and uses more molecular oxygen to generate superoxide, thereby leading to the formation of peroxynitrite (PNT). Thus, the coexpression of arginase and NOS and the subsequent production of PNT in subsets of myeloid cells like myeloid-derived suppressor cells (MDSCs) could be the result of an uncoupled NOS. Moreover, increased arginase expression can limit NOS2 expression in immune cells by decreasing the arginine needed for NOS2 translation (43).

## Arginine Metabolism in Macrophages

The concept of basing macrophage activation into M1 and M2 subsets with distinct functional consequences on the usage of arginine *via* NOS or arginase has been described several decades ago. M1 and M2 macrophages induce Th1 and Th2-like inflammatory responses that further intensify M1- and M2-type responses, respectively (44, 45). Stimulation of bone marrow-derived or peritoneal inflammatory macrophages with TLR agonists activates transcription factors such as nuclear factor kappa-light-chain-enhancer (NF-κB) that induce pro-inflammatory cytokines like IFNγ resulting in NOS2-expressing M1 macrophages, while cytokines like IL-4 and IL-13 activate STAT6 and lead to arginase 1-expressing M2 macrophages (46–49). Arginase 2, however, is not significantly modulated by Th1 or Th2 cytokines (4). Due to the fact that the *in vitro* culture systems do not precisely represent the multiple potential factors that affect macrophages *in vivo* as well as the discrepancies in the mouse and human results, the phenotypic and functional aspects of macrophage polarization remain to be tackled (50–52).

### Arginine Metabolism in Classically Activated Macrophages

M1 macrophages, *via* NOS2, metabolize arginine to nitric oxide and citrulline (8, 14). Besides its multiple signaling pathways (9, 12), the cytotoxic properties of nitric oxide make M1 macrophages well suited to function as key effector cells for the elimination of intracellular pathogens and tumor cells. When induced, M1 macrophages likely use all imported arginine, and NOS2 generates nitric oxide in large quantities, also indicating the importance of arginine availability and uptake as a rate-limiting step for nitric oxide synthesis (53). To more efficiently produce nitric oxide, or when arginine is limited, citrulline is reused to synthesize nitric oxide *via* the so-called citrulline–nitric oxide cycle. These reactions involve two enzymes: argininosuccinate synthase (ASS1) and argininosuccinate lyase (ASL) (Figure 1). While ASS1 can be induced by TLR agonists and IFNγ, M1 macrophages constitutively express ASL (54, 55). In fact, mice lacking ASS1 fail to control mycobacteria infection, therefore confirming the importance of citrulline recycling *via* ASS1 and ASL in nitric oxide production by M1 macrophages (56).

Excessive nitric oxide synthesis can lead to unwanted host cytotoxicities and imbalanced immune responses. Upregulation of arginase 1, however, is a means by which macrophages limit the availability of arginine and regulate nitric oxide production (57). Besides the fact that the availability of arginine may control the translation of NOS2 mRNA (43), polyamines, which restrict the immune effector function of macrophages in response to TLR agonists (58, 59), also inhibit the cationic amino acid transporter 2B (CAT-2B) arginine transporter and nitric oxide synthesis in macrophages (60). Consistent with this, polyamines diminish *Helicobacter pylori*-induced NOS2 protein levels and nitric oxide production in macrophages through a post-transcriptional effect on NOS2 translation, whereas ODC inhibition enhances NOS2 protein expression and macrophage nitric oxide-dependent killing of bacteria (61). Another mechanism underlying the upregulation of arginase 1 in M1 macrophages involves a TLR-myeloid differentiation primary response 88 (MyD88)-dependent pathway. TLR signaling in mycobacteria-infected macrophages stimulates the production of cytokines like IL-6, IL-10, and granulocyte colony-stimulating factor (G-CSF) that provoke arginase 1 expression in an autocrine–paracrine fashion, involving the transcription factors STAT3 and C/EBPβ (62).

Given the complexity of inflammatory cues within diseased tissue microenvironments, it is essential to consider the interrelated and dichotomous regulation of macrophage arginine metabolism in determining the type and outcome of immune response against pathogens. For instance, in a model of *Leishmania major*, TNFα mediates protection by restraining the development of arginase 1-expressing M2 macrophages and dendritic cells (DCs), while maintaining the production of nitric oxide *in situ* (63). Other disease models, however, show different interactions between NOS2 and arginase 1. In tuberculosis granulomas, arginase 1-expressing M2 macrophages localize to the outer regions of granulomas, while NOS2-expressing M1 macrophages can be found in the inner regions. This provides an organized microenvironment within granulomas that separates anti-microbial (M1, NOS2-mediated) and anti-inflammatory (M2, arginase 1-mediated) responses to constraint lung pathology (64). In addition to its role in regulating NOS2 activity through arginine competition, arginase 1 also regulates T cell proliferation, thereby playing a significant role in the control of *Mycobacterium tuberculosis* growth and pathology independently of NOS2 suppression (65). Conversely, when *M. tuberculosis*-infected mice are coinfected with *Schistosoma mansoni*, arginase 1-expressing M2 macrophages expand and limit the microbicidal immune response, thus increasing the disease progression and severity (66). Such findings clearly point out to the significance of the signals within *in vivo* immune milieus that can instruct macrophages and can often not be uncovered through *in vitro* evaluation.

### Arginine Metabolism in Alternatively Activated Macrophages

Arginase 1-expressing M2 macrophages play pivotal roles in multiple immunopathological settings. M2 macrophages regulate immune responses mostly through redirecting arginine away from NOS with arginase 1 or *via* ornithine production. Indeed, macrophages producing arginase 1 and ornithine have widely been considered critical for wound healing (67, 68). These results were recently confirmed using pharmacologic inhibition of arginase and, more importantly, macrophage-specific arginase 1 knockout mice (69). The decrease in arginase 1 expression results in a heightened infiltration of NOS2-expressing cells, reduced matrix deposition, and delayed healing (69).

M2 macrophage-specific arginase 1 blocks inflammation and fibrosis post infection with *S. mansoni*. For instance, conditional deletion of arginase 1 in macrophages is associated with accelerated mortality due to uncontrolled Th2 cytokine-induced pathology in the livers of infected mice (70). Although early studies of schistosomiasis pathology indicate that M2 macrophage arginase 1 might contribute to liver fibrosis *via* production of proline as a precursor of collagen (46), mice lacking arginase 1 in macrophages display increased liver fibrosis and collagen deposition (70). Likewise, macrophage-derived arginase 1 is protective against excessive injury of the intestinal tissue of infected mice (71). In addition to suppressing T cell proliferation, arginase 1 enhances regulatory T (Treg) cell and limits Th17 cell phenotype; furthermore, arginase 1 deficiency in macrophages results in IL-12/IL-23p40-dependent neutrophil-linked gut pathology (71). On the contrary, macrophage-specific arginase 1 is not essential in multiple murine models of Th2 lung inflammation and asthma (72), suggesting that the regulatory outcomes of macrophage-derived arginase 1 are organ specific. Although not technically supported, it is hypothesized that the function of arginase 1 varies according to the relative rate of perfusion, and hence ultimately arginine availability, in different organs (72). Nevertheless, other studies indicate that subsets of arginase 1-producing M2 macrophages may serve a role in allergic immune responses (73), indicating that this still is an exciting area for future investigation.

Macrophage arginase 1 also directly controls parasite growth. In a murine model of *Heligmosomoides polygyrus*, memory CD4^+^ T cells produce IL-4, therefore recruiting M2 macrophages that block larval parasite health and mobility *via* an arginase 1-dependent mechanism (74). Additionally, *H. polygyrus*-specific antibodies and helminth larvae induce arginase 1 expression in macrophages independent of IL-4Rα signaling, and the arginase 1 product ornithine directly inhibits larval motility (74). With this result, antibodies represent a novel pathway of macrophage alternative activation throughout Th2 immune responses.

## Arginine Metabolism in DCs

Dendritic cells are the most professional antigen-presenting cells known as key mediators between innate and adaptive immune responses. They present pathogen-derived antigenic peptides and provide costimulatory molecules and cytokines crucial for T cell activation and differentiation (75, 76). As such, how properly DC function is regulated largely shapes T cell antigen-specific immunity in different disease scenarios. DCs are classified into several subpopulations with distinct phenotypes, functions, and locations. These include but are not limited to conventional DCs, monocyte-derived DCs, and plasmacytoid DCs (77, 78). The enzymes involved in arginine metabolism, NOS2 and arginase 1, are implicated in the function of subsets of DCs that evolve in response to local environmental stimuli. Similar to macrophages, the differential expression of these enzymes results in dichotomous functions within these critical immune cells.

A population of TNFα and iNOS-producing DCs (Tip-DCs) has recently been described (79, 80). These cells are characterized as CD11b^+^CD11c^+^Ly6C^+^MHC-II^+^. The initial reports indicate that Tip-DCs can mediate the resistance to pathogens such as *Listeria* (79), *Brucella* (81), and *Leishmania* (82). However, Tip-DCs can also contribute to the liver pathogenicity in *Trypanosoma brucei*-infected mice; in this context, TNFα and nitric oxide production is IFNγ and MyD88 signaling dependent (83). Moreover, Tip-DCs can interact with tumor-infiltrating antigen-specific CD8^+^ T cells to mediate tumor growth rejection (84). In this model, tumor antigen-reactive CD8^+^ T cells activate Tip-DCs that in turn present tumor-associated antigens, thereby enhancing T cell expansion and tumor killing *via* TNFα and nitric oxide production. While colony-stimulating factor 1 receptor (CSF-1R) signaling is not required, CD40–CD40L signaling is a key pathway for nitric oxide production and the antitumor response (84). Thus, activated T cells carry the possibility to modulate the inflammatory tumor microenvironment despite the fact that they are also targets of the suppressive elements of the same milieu, suggesting the tight balance occurring in tumors.

Tumors, however, can educate DCs to acquire an immunosuppressive phenotype, represented by low costimulatory molecule expression, poor antigen presentation, and high expression of regulatory receptors (85). Murine lung cancer-derived TGFβ and PGE2 favor the generation of tolerogenic DCs (CD11b^high^CD11c^low^MHC-II^low^) that inhibit the proliferation of CD4^+^ T cells *in vitro* and *in vivo* (86). Arginase 1, induced mainly by PGE2, plays a significant role in this effect (86). IL-6 also promotes the expression and activity of arginase 1 that subsequently downregulates MHC-II in DCs and suppresses CD4^+^ T cell-mediated antitumor immunity (87). Although murine spontaneous mammary tumors are infiltrated with phenotypically mature DCs (CD11b^+^CD11c^high^MHC-II^high^), these DCs suppress CD8^+^ T cell function *via* arginase 1 production, thus leading to impaired T cell antitumor immunity (88).

## Arginine Metabolism in MDSCs

Myeloid-derived suppressor cells are associated with several immune regulatory aspects in conditions involving chronic inflammation, such as cancer (89, 90), infections (91–93), trauma (94), obesity (95, 96), graft versus host disease (97), and autoimmune diseases (98, 99). With a sustained status of abnormal myelopoiesis, MDSCs represent a heterogeneous population of myeloid progenitor cells distinct from mature myeloid cells like macrophages, DCs, and neutrophils. MDSCs potently suppress innate and adaptive immunity and comprise two major subsets, namely, monocytic MDSCs (M-MDSCs) and polymorphonuclear MDSCs (PMN-MDSCs). In mice, M-MDSCs are CD11b^+^Ly6C^high^Ly6G^−^, while PMN-MDSCs are CD11b^+^Ly6C^low^Ly6G^+^. In humans, M-MDSCs are defined as CD33^+^CD14^+^CD15^−^HLA-DR^low^, while PMN-MDSCs are CD33^+^CD14^−^CD15^+^HLA-DR^−/low^ or CD33^+^CD14^−^CD66b^+^HLA-DR^−/low^ (100).

Multiple tumor-associated factors drive MDSC accumulation and acquisition of immunosuppressive function. For instance, vascular endothelial growth factor is associated with an arrest in DC maturation, while concomitantly expanding MDSCs, through the inhibition of NF-κB signaling (101, 102). Whereas G-CSF plays a critical role in mobilizing and differentiating bone marrow granulocytic precursors within tumors (103), GM-CSF, depending on the stimulation magnitude and context, promotes MDSC accumulation *in vitro* and *in vivo* (104, 105). IL-1β is also a potent driver of MDSCs either directly or indirectly through stimulating other mediators such as IL-6 (106, 107). IL-4 and IL-13 evoke MDSC suppressive mechanisms *via* IL-4Rα-dependent STAT6 activation (108, 109). Other pro-inflammatory danger signals secreted mostly by myeloid cells such as S100A8/A9 proteins and high-mobility group box 1 can also enhance MDSC trafficking and function by signaling through cell membrane receptors like TLRs and the receptor for advanced glycation end-products (110, 111). Similarly, several transcription factors are critical for MDSCs. Among those, STAT1 activated by type 1 and 2 IFNs and IL-1β drives MDSC accumulation and regulatory mechanisms (NOS2 and arginase 1) (112, 113). The induction of STAT3 and STAT5 *via* G-CSF and GM-CSF, respectively, downregulates IFN regulatory factor 8 that essentially drives MDSC accumulation (114). STAT3 can also induce genes important for MDSC differentiation and proliferation (c-myc, cyclin D1, and S100A8/A9) and suppressive function (NADPH oxidase [NOX] subunits p47^phox^ and gp91^phox^ and C/EBPβ) (115–118). C/EBPβ-homologous protein (Chop) induced by tumor-derived reactive oxygen species (ROS) and reactive nitrogen species (RNS) and regulated by activating-transcription factor 4 promotes IL-6 production and activates C/EBPβ as well as STAT3, thereby mediating MDSC regulatory function (119).

Myeloid-derived suppressor cells promote immune dysfunction using different mechanisms, either directly *via* depriving T cells of essential metabolites such as arginine, tryptophan, and cysteine or interfering with T cell viability, migration, or activation or indirectly *via* inducing other immune regulatory cells such as Treg cells and tumor-associated macrophages (TAMs) (89, 90, 120). Depletion of arginine through arginase 1 is one of the first T cell suppressive mechanisms described in MDSCs. PMN-MDSCs, the major source of arginase 1 in tumor-bearing hosts, reduce extracellular arginine by arginine incorporation *via* CAT-2B or arginase 1 production (3, 121). Arginase 1 inhibitors such as N^ω^-hydroxy-nor-arginine (nor-NOHA) or N^ω^-hydroxy-arginine (NOHA) block MDSC suppressive activity and result in an immune-mediated, dose-dependent T cell antitumor immunity (3, 90). Starving T cells of arginine downregulates the expression of CD3ζ, a hallmark of T cell dysfunction in cancer patients (122). However, arginine-starved T cells produce IL-2 and upregulate the early activation markers CD25, CD69, and CD122, indicating that the effect induced by arginine depletion is not due to a defect in T cell receptor (TCR) signaling (123). On the other hand, arginine-starved T cells are arrested in the G0–G1 phase of the cell cycle as a result of an impaired expression of cyclin D3 and cyclin-dependent kinase 4 (cdk4) in T cells through a decreased mRNA stability and diminished translational rate (124, 125). Interestingly, *in vivo* deprivation of arginine impairs T cell responses due to a general control non-derepressible 2 (GCN2) kinase-dependent accumulation of MDSCs (126). More recently, arginine has been found to be critical for T cell metabolic fitness and survival, and therefore, increasing the intracellular arginine abundance in T cells prior to adoptive cellular therapy (ACT) enhances their persistence and antitumor responses (36). In addition to the high susceptibility to low extracellular arginine, T cells also fail to respond in environments that lack cysteine or in those having indoleamine 2,3-dioxgenase (IDO)-mediated tryptophan deprivation (127, 128). A potential role of the integrated stress responses has been suggested as a common mediator of the effects induced by amino acid deprivation (124, 129). Briefly, accumulation of empty aminoacyl-tRNAs caused by low amino acid content activates GCN2, which phosphorylates the eukaryotic translation initiation factor 2α (eIF2α). The phosphorylated form of eIF2α binds with higher affinity to eIF2β, blocking its ability to exchange guanosine diphosphate (GDP) for guanosine triphosphate (GTP), which then inhibits the binding of the eIF2 complex to methionine aminoacyl-tRNA. This results in a decreased initiation of global protein synthesis. Accordingly, culture of cells in the absence of arginine induces a significant phosphorylation of eIF2α and global decrease in protein synthesis (130). In addition, T cells from GCN2 knockout mice display a lower susceptibility to amino acid availability (129). Recent studies also point out to the essential role of rapamycin-insensitive companion of mammalian target of rapamycin (Rictor)/mTOR complex 2 in regulating the responses induced by limiting amino acids (131). As such, T cells lacking Rictor/mTOR are resistant to amino acid starvation-induced immunosuppression (132, 133), suggesting the relevance of this pathway in the suppression of T cell responses by amino acid depletion.

Myeloid-derived suppressor cells also exert their immunosuppressive effect through nitric oxide production by NOS2 in M-MDSCs and NOS3 in PMN-MDSCs (134). In addition to its direct apoptotic effects, nitric oxide negatively regulates T cells by impairing the IL-2R signaling pathways Jak-3, STAT5, ERK, and AKT (135, 136). Furthermore, MDSCs *via* the NOX subunits p22^phox^, p47^phox^, and gp91^phox^ produce ROS such as superoxide and hydrogen peroxide that inhibit T cell CD3ζ expression and cytokine production (116). Nitric oxide can then react with superoxide to produce more detrimental RNS such as PNT generated by PMN-MDSCs depending on the expression of gp91^phox^ and NOS3 (134). PNT can induce T cell apoptosis *via* the nitration of tyrosine residues, thereby blocking protein tyrosine phosphorylation (137). PNT also disrupts the conformational flexibility of the TCR-MHC/peptide binding by nitrating/nitrosylating the TCR and MHC, thus limiting T cell antitumor immunity (138, 139). Moreover, PNT hinders the infiltration of T cells, while facilitating the trafficking of MDSCs, into tumors mostly through the nitration of chemokines such as CCL2 and CCL5 or chemokine receptors such as CXCR4 (140, 141).

## Arginine Methylation in Inflammation

Posttranslational methylation of arginine residues in proteins through the protein arginine methyltransferases (PRMTs) regulates multiple cellular signaling pathways related to cell differentiation, proliferation, and function. Arginine methylation by PRMTs activates or inhibits multiple transcription factors and other proteins, thereby regulating chromatin remodeling, RNA splicing, DNA damage repair, and protein–protein interactions. These events occur through the formation of aDMA, made by type I PRMTs, symmetric dimethylarginine (sDMA), made by type II PRMTs, or monomethylarginine (MMA), made by type III PRMTs. The major PRMTs associated with the regulation of immunity include the coactivator associated arginine methyltransferase 1 (CARM1 or PRMT4), PRMT1, PRMT5, and PRMT6. CARM1 targets proteins regulating chromatin remodeling and RNA-binding proteins (142). In addition, CARM1 is a major coactivator of NF-κB (143). Similarly, PRMT1 has been recognized as a major regulator of inflammation through its ability to methylate multiple proteins, including NF-κB (144), CITED2, STAT5 (145), and NFAT (146). Furthermore, PRMT5 and PRMT6 have been shown to increase the activity of NF-κB, thereby regulating the expression of IL-1α and IL-6 (147, 148). Although the capacity of arginine methylation to modulate multiple inflammatory signaling pathways has been described, its role in several pathologies and especially in patient populations remains to be investigated.

## Therapeutic Implications and Concluding Remarks

Arginine deprivation is a novel therapeutic modality for several ASS1-deficient, arginine-auxotrophic solid and hematological malignancies. To this end, arginine-metabolizing enzymes, mycoplasma-derived arginine deiminase (ADI) and recombinant human arginase 1, have been pegylated for enhanced *in vivo* pharmacokinetics and pharmacodynamics (149, 150). Pegylated ADI (peg-ADI) controls tumor growth in multiple xenograft models, including melanoma, hepatocellular carcinoma, and lung cancer (151, 152). Promoting apoptosis and blocking angiogenesis and *de novo* protein synthesis are endorsed antitumor mechanisms for peg-ADI treatment (149, 150). Clinical investigations of peg-ADI have followed in melanoma and hepatocellular carcinoma, with response rates of 25 and 47%, respectively (153, 154). Several other phase II and phase III clinical trials are underway testing peg-ADI in patients with metastatic melanoma, advanced hepatocellular carcinoma, and small-cell lung cancer. With the clinical evidence for developing anti-peg-ADI neutralizing antibodies (155, 156), recombinant human arginase 1 represents an alternative arginine deprivation therapy. Pegylation extends the half-life of arginase 1 without altering its activity (157). Pegylated arginase 1 (peg-arginase 1) is effective against several cancers, such as melanoma, hepatocellular carcinoma, and leukemia (130, 157, 158). The antitumor effect of peg-arginase 1 is mediated *via* the induction of autophagy, apoptosis, and cell cycle arrest in malignant cells (130, 150, 159). Peg-arginase 1 is currently undergoing clinical investigation in patients with advanced hepatocellular carcinoma. The initial clinical evidence indicates that peg-arginase 1 is safe and results in prolonged arginine depletion (160). Interestingly, peg-arginase 1 also induces immune suppression by reducing the availability of arginine to primary T cells and through the induction of MDSCs (126). As such, peg-arginase 1 extends the survival of mice undergoing bone marrow transplantation and delays the appearance of graft versus host diseases, whereas enhancing the growth of *Listeria* (97, 161). Additionally, peg-arginase 1 exerts a potent anti-herpetic activity, blocking herpes simplex virus replication and virus-derived cytopathic effects *in vitro* (162). Arginine-catabolizing enzymes have a preclinical additive and/or synergistic effects with other treatments such as chemotherapy, radiotherapy, PI3K inhibitors, and autophagy regulators (149), and the clinical efficacy of these combinatorial approaches remains to be determined.

As outlined above, the arginine-metabolizing enzymes arginase 1 and NOS2 are key suppressive mechanisms by which immunoregulatory myeloid cells restrain T cell antitumor immunity (Figure 2), thus indeed paving the way for developing strategies to target these pathways. The arginase 1 inhibitor nor-NOHA and the ODC inhibitor α-difluoromethylornithine (DFMO) downregulate arginase 1 expression in tumor-associated MDSC and restore T cell antitumor immunity (3, 163). Since arginase 1 expression can be driven by cyclooxygenase 2 (COX2)/PGE2 axis, celecoxib, a selective COX2 inhibitor, also blocks arginase 1 expression, reduces MDSC accumulation, and elicits CD4^+^ and CD8^+^ antitumor immune responses (30). Moreover, dietary celecoxib synergizes with DC-based vaccination to extend the survival of mesothelioma-bearing mice (164). While these studies have examined the role of MDSCs, it is conceivable that these agents could also modulate other arginase 1-expressing myeloid cells like TAMs and tolerogenic DCs (85, 165). On the other hand, *N*(6)-(1-iminoethyl)-l-lysine-dihydrochloride (l-nil), a NOS2 selective inhibitor, constraints melanoma growth and improves the survival of tumor-bearing mice, and a combination of l-nil and cisplatin is better than either agent alone (166). Reports, however, recommend targeting both arginase 1 and NOS2 to augment the therapeutic effect. In a model of human prostatic adenocarcinomas, only concomitant inhibition of arginase 1 and NOS2 reduces PNT production and recovers tumor-infiltrating lymphocyte antitumor responsiveness (167). Phosphodiesterase-5 (PDE5) inhibitors (sildenafil, tadalafil, and vardenafil) decrease the expression of arginase 1 and NOS2, thereby blocking MDSC regulatory activity (168). Accordingly, PDE5 inhibitors promote intratumoral infiltration of activated T cells, control tumor growth, and enhance the efficacy of ACT (168). Likewise, nitric oxide-releasing aspirin (a typical aspirin linked to a nitric oxide donor) reduces arginase 1, NOS2, and PNT, while increasing the frequency and function of tumor-specific T cells, thus boosting the antitumor effect of cancer vaccination (169). Another small molecule that prevents PNT production *in vivo*, namely, 3-[(aminocarbonyl)furoxan-4-yl]methyl salicylate (AT38), also drives the infiltration of tumor antigen-specific T cells into tumors and synergizes with ACT (141). The fact that agents like PDE5 inhibitors and nitric oxide-releasing aspirin have been proven safe in patients provides a rationale to use these treatments in combination with other immunotherapeutic approaches such as ACT and checkpoint blockade. Additionally, as discussed above, NOS2-expressing Tip-DCs are critical for tumor rejection in the context of ACT in mice (84). Of interest, this antitumor response does not require lymphodepletion preconditioning prior to ACT. Therefore, this development could reprogram the immunosuppressive tumor microenvironment and, more importantly, obviate the need for other potentially toxic regimens.

Overall, arginine metabolism has evolved as a key player in the center of our immune system. At this point, it is very clear that the regulators of arginine metabolism can elicit dichotomous innate and adaptive immune responses for instance in controlled versus uncontrolled infection, autoimmunity versus self-tolerance, and antitumor immunity versus tumor-induced immune suppression. As such, a better understanding of arginine metabolic pathways within the complicated inflammatory microenvironments *in vivo* and in the human as opposed to the mouse system will facilitate the development of targeted therapeutic interventions in different diseases.

## Author Contributions

PR wrote and critically revised the manuscript. AO discussed the manuscript. AAA wrote and critically revised the manuscript.

## Conflict of Interest Statement

The authors declare that the research was conducted in the absence of any commercial or financial relationships that could be construed as a potential conflict of interest.
